# Bioethanolic yeasts from dung beetles: tapping the potential of extremophilic yeasts for improvement of lignocellulolytic feedstock fermentation

**DOI:** 10.1186/s13068-021-01940-y

**Published:** 2021-04-07

**Authors:** Anita Ejiro Nwaefuna, Karl Rumbold, Teun Boekhout, Nerve Zhou

**Affiliations:** 1 Department of Biological Sciences and Biotechnology, Botswana International University of Science and Technology, Private Bag 16, Palapye, Botswana; 2 Industrial Microbiology and Biotechnology Laboratory, School of Molecular and Cell Biology, University of the Witwatersrand, Johannesburg, South Africa; 3 Westerdijk Fungal Biodiversity Institute, 3584CT Utrecht, the Netherlands; 4 Institute for Biodiversity and Ecosystem Dynamics (IBED), University of Amsterdam, 1098 SM Amsterdam, the Netherlands

**Keywords:** Bioethanol production, Dung beetles, Yeasts, Fermentation, Extremophilic trait

## Abstract

Bioethanol from abundant and inexpensive agricultural and industrial wastes possesses the potential to reduce greenhouse gas emissions. Bioethanol as renewable fuel addresses elevated production costs, as well as food security concerns. Although technical advancements in simultaneous saccharification and fermentation have reduced the cost of production, one major drawback of this technology is that the pre-treatment process creates environmental stressors inhibitory to fermentative yeasts subsequently reducing bioethanol productivity. Robust fermentative yeasts with extreme stress tolerance remain limited. This review presents the potential of dung beetles from pristine and unexplored environments as an attractive source of extremophilic bioethanolic yeasts. Dung beetles survive on a recalcitrant lignocellulose-rich diet suggesting the presence of symbiotic yeasts with a cellulolytic potential. Dung beetles inhabiting extreme stress environments have the potential to harbour yeasts with the ability to withstand inhibitory environmental stresses typically associated with bioethanol production. The review further discusses established methods used to isolate bioethanolic yeasts, from dung beetles.

## Background

### Bioethanol and bioethanolic yeasts: gaps, limitations, and challenges

**Fig. 1:**
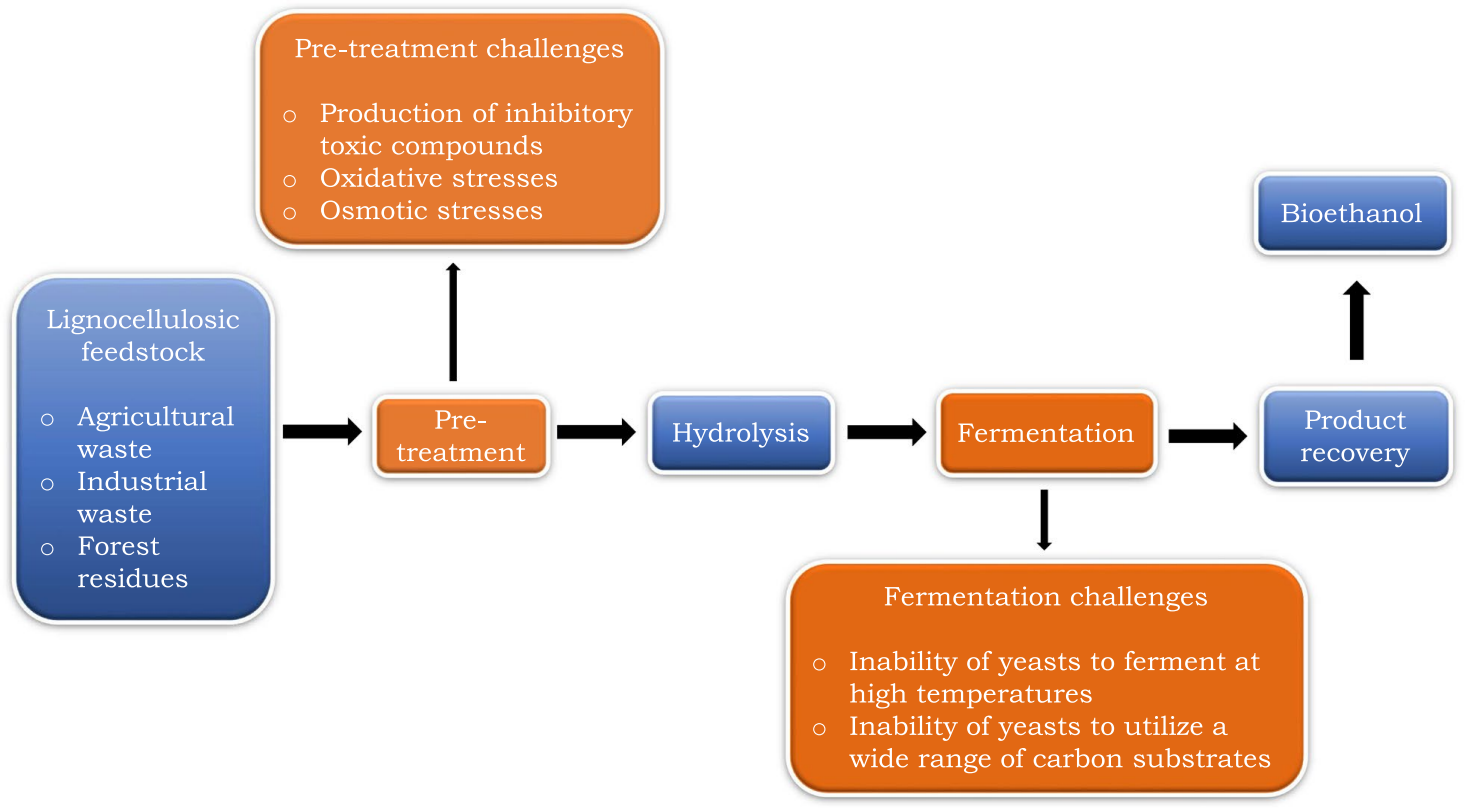
An overview of challenges affecting bioethanol production in the pre-treatment and fermentation stages

Bioethanol is an attractive alternative to petroleum fuels due to its numerous advantages [1–7]. Reduced reliance on non-renewable energy sources and a decrease in greenhouse gas emissions are the two major merits. Although fossil fuels meet our demand for energy, their contribution to climate change as a result of greenhouse gas emissions is a global dilemma. The transportation sector is the world’s biggest contributor to greenhouse gas emissions due to its high dependence on fossil fuels [8–11]. The use of renewable fuel in this sector to achieve greenhouse gas emission neutrality is an imminent solution for sustainable and environmentally friendly energy sources. However, for bioethanol to be competitive against non-renewable fuel sources, there are many hurdles to overcome [11–13]. One of the most important hurdles is food security. First-generation bioethanol produced from edible food crops such as corn, rice, barley, potatoes, sugarcane, and other starchy food crops [14–18] has led to controversies due to competition with food production and land use. The competition for land use increases the demand and price of food crops [19–23]. Second-generation bioethanol using non-edible, inexpensive, and abundant lignocellulosic biomass such as woody and herbaceous biomass, forest residues, industrial and agricultural wastes, and other non-food crops [16, 18, 24] as raw materials address the food versus fuel challenge associated with the use of food-grade feedstocks. Although second-generation bioethanol processing generates far lower levels of greenhouse gases as compared to the first-generation alternative [25], it is not without demerits. Production costs associated with separate hydrolysis and fermentation (SHF) processes where each step is carried out under optimal pH and temperature conditions [26] are extremely high to achieve sustainability. Recently, however, Simultaneous Saccharification and Fermentation (SSF) is a method that has become increasingly attractive in the production of bioethanol from non-edible lignocellulosic feedstocks, as well as dedicated energy crops [13, 27, 28] as it produces higher bioethanol yields than SHF [2, 7, 29–31]. This method allows hydrolysis and fermentation undertaken simultaneously to reduce production costs. Lignocellulosic feedstocks are pre-treated using physical, chemical, and biological pre-treatments [32, 33]. Chemical pre-treatment exploiting either acid or alkali treatments [34–37] at high temperatures is the most preferred method. However, this pre-treatment process creates another hurdle, because there are limited robust yeasts strains that can tolerate the presence of inhibitory toxic compounds such as acetic acids, levulinic acids, furfural, 5-hydroxymethylfurfural (HMF), and ferulic acids [7, 16, 20, 38, 39] which are generated during this process (Fig. 1). In addition, the process exerts extreme osmotic and oxidative stresses [19, 32, 40, 41] (Fig. 1), which are extremely harsh for the conventional bioethanolic yeasts. These conditions can inhibit or reduce speed of growth and fermentation efficiency of yeasts [16, 17, 28, 36]. The use of biological treatments, such as thermotolerant cellulolytic enzymes (optimal temperature 45–80 °C) [42], is another option. One major drawback is the wide difference of temperature requirements for this treatment and those used for growth and fermentation of yeasts which ranges from 20 to 35 °C [26] if the process is to be simultaneous as a cost-cutting measure. Therefore, using fermentative yeasts with extreme stress tolerance attributes such as thermotolerance, ethanol tolerance, oxidative stress tolerance, osmotolerance, and tolerance to inhibitory substrates among others would be ideal to overcome these drawbacks. Currently, mesophilic yeasts are used to produce bioethanol. The most desirable yeasts are thermophiles fermenting at temperatures of 40 °C or higher as this reduces the costs of pumping and cooling as well as allowing for efficient saccharification [43]. Efficient saccharification subsequently increases the amount of available fermentable sugars, which improves the overall fermentation productivity. In addition, higher temperature fermentation minimises contamination risks among other advantages. Examples of thermophilic yeasts such as *Issatchenkia orientalis* [44] and *Kluyveromyces marxianus* [45] are ideal, although they have their limitations such as lower ethanol yield from lignocellulosic feedstocks, poor tolerance to inhibitors and ethanol leading to a lower fermentation efficiency [46]. Hydrolysis of lignocellulosic feedstocks, which typically consists of cellulose, hemicellulose, and lignin, yields various fermentable sugars, such as pentoses (for example, xylose and arabinose) and hexose sugars (for example, mannose and galactose) [1, 16, 19]. A wide substrate utilization range is an essential characteristic of bioethanol production yeasts. The wild-type conventional/traditional yeast used in bioethanol fermentation, *Saccharomyces cerevisiae*, is incapable of fermenting pentose sugars, which subsequently reduces the efficiency of the process (Fig. 1). For example, this yeast cannot ferment xylose, the most abundant pentose sugar in lignocellulosic feedstocks [3]. The search for extremophilic and robust fermentative yeasts from dung beetles inhabiting extreme environments is one way to progressively increase productivity and subsequently the sustainability of bioethanol.

Dung beetles of the *Scarabaeidae* family and order *Coleoptera* are dependent on lignocellulose-rich animal dung for survival and reproduction [47, 48]. About 80% of dung is composed of indigestible carbon sources such as cellulose, tannin, and lignin with low nutritional content and quality [49–51]. Such a recalcitrant material for a diet suggests that symbiotic associations are obligatory for dung beetles to efficiently process lignocellulose within their guts. Such a diet requires immense specialisation for resident yeasts, which could serve as a unique ecological niche for novel yeasts with the ability to degrade inexpensive lignocellulosic feedstocks in bioethanol production processes. For decades, dung beetle research suggests that their guts possess microbial consortia, including yeasts which provide nutrition and assist in the digestion of such complex substrates [49]. The search for yeasts from dung beetles could be extended to those beetles inhabiting harsh environments like the hot deserts and tropical surroundings as extremophilic environments. Such ecological niches could be even more attractive as a source of multi-stress tolerant yeasts, which are likely to be found in association with resident extremophilic beetles. Robust stress tolerance is a phenotype of interest for the techno-economic feasibility of bioethanol production. Stressors such as weak acids and furans generated by hydrolysis of lignocellulosic feedstocks, ethanol, and high-temperature fermentation severely reduce the fermentative capacity of the bioethanolic yeasts.

This review presents an overview of challenges encountered during bioethanol production by conventional yeasts and the potentiality of dung beetles in harbouring novel yeasts with various attributes beneficial to the bioethanol industry.

### Dung beetle as a source of extremophilic and ethanologenic yeasts

Symbiotic relationships between phylogenetically diverse insects and yeasts are well documented [52–55]. Insects mutually benefit from an association with yeasts; by attraction to food, whereas yeasts are vectored by insects from one food substrate to another [53, 54, 56]. Yeasts have a role to play in the nutrition of beetles, for example, secretion of enzymes for digestion of food [57–59], provision of essential organic acids, vitamins, as well as other products of their carbon metabolism pathway [58, 60, 61]. Well-documented examples of yeasts associated with *Drosophilid* insects suggest that yeasts provide essential vitamins (e.g., thiamin) and lipids needed for metabolism, and also assist with their reproduction by facilitating chemical communications and mating activities [54, 58, 62]. The growth and development of insects colonies such as honeybees are enhanced by yeasts inhabiting their gastrointestinal tract [54]. Yeasts found in termites play a role in the digestion of wood by producing relevant enzymes for xylose degradation [54, 63]. Furthermore, yeasts help to regulate various interactions among insect species. Yeasts are vectored by insects from one environment to another and are protected from adverse environments as well as resource depletion [53, 54, 58], as compared to their microbial counterparts, bacteria, and fungi, which can be dispersed through air; for example, flower beetle insects vector yeasts (e.g., *Kuraishia capsulata* and *Yamadazyma tenuis*) from one flower to another [54]. The environment within the insect gastrointestinal tract is conducive for yeast growth, reproduction, and survival. Yeasts inhabiting beetle intestines are provided with xylose sugar therefore enabling such yeasts to acquire xylose utilization and fermentative traits.

Yeast biodiversity in many insects like dung beetles for industrial applications has been poorly studied. About 1% of yeasts species is currently known and more are yet to be discovered [64]. Over 650 yeasts have been isolated from beetle guts [65]. *Candida sp, Hanseniaspora sp, Kluyveromyces sp, Metschnikowia sp, Pichia sp,* and *Saccharomyces sp* [65] are examples of yeast genera isolated from beetle guts that can be used for lignocellulosic feedstocks fermentations. A novel yeast *Trichosporon heliocopridis* *sp. nov* isolated from a dung beetle (*Heliocopris bucephalus* Fabricius) [66] was reported to assimilate a variety of carbon sources like glucose, sucrose, galactose, maltose, raffinose, trehalose, D-arabinose, and lactose to mention a few. The yeast was incapable of assimilating other carbon sources such as cellobiose, soluble starch, melibiose, D-xylose, L-arabinose, and L-sorbose. A major downside to this is that the yeast could not ferment glucose. However, the yeast’s ability to ferment other sugars, as well as its thermotolerance ability are ideal background traits for strain development for example via evolutionary engineering [67]. Evolutionarily engineering of *Candida intermedia,* for example*,* enhanced xylose conversion, production of ethanol, and tolerance to lignocellulose-derived compounds [68]*.* Exploring robust fermentative yeasts capable of utilizing inexpensive and abundant carbon sources as well as exhibiting extremophilic stress tolerance traits from dung beetles that inhabit extreme and pristine environments could tremendously improve the economic feasibility of bioethanol.

Other than the ability to ferment multiple sugars, stress tolerance is an essential attribute for bioethanol fermenting strains. Yeasts from dung beetles inhabiting extreme environments with abilities to withstand stresses such as high temperatures, high salt, sugar, and ethanol concentrations could be isolated. Examples of such traits are listed in Table 1.

**Table 1:** Possible yeasts with extremophilic traits for efficient bioethanol production

Trait	Yeast species	References
High ethanol productivity	*S. cerevisiae*	[98]
	*Brettanomyces* (= *Dekkera*) *bruxellensis*	[85, 99, 100]
	*K. marxianus*	[45, 101, 102]
Lignocellulosic-based carbon (e.g., cellulose, arabinose, xylose)	*P. stipitis, C. shehatae, and P. tannophilus, Schizosaccharomyces spp*	[103]
Thermotolerance	*K. marxianus*	[13, 18, 32, 86–88, 104–106]
	*O. polymorpha*	[87–91]
	*P. kudriavzevii*	[86, 107–113]
Osmotolerance	*Z. rouxii*	[86, 88, 91, 114–116]
	*Torulaspora delbrueckii*	[86, 116, 117]
	*Z. bailii*	[91, 115, 117, 118]
	*Metschnikowia pulcherrima*	[86]
	*Wickerhamomyces anomalus*	[24, 86, 119]
	*Debaryomyces hansenii*	[86, 117, 120]
	*Schizosaccharomyces pombe*	[86, 115, 117]
	*P. kudriavzevii*	[117]
Halotolerance	*Z. rouxii*	[88, 115, 116]
	*T. delbrueckii*	[86, 88]
	*W. anomalus*	[86, 88, 115]
	*D. hansenii*	[86, 88, 106, 115, 117]
Ethanol tolerance	*O. polymorpha*	[87, 89–91, 121]
	*W. anomalus*	[86]
	*D. bruxellensis*	[86, 91, 115, 122]
	*S. pombe*	[115]
	*T. delbrueckii*	[86, 123]
	*Z. bailii*	[86, 115, 120]
Furan derivative tolerance	*P. kudriavzevii*	[86, 91, 124, 125]
	*W. anomalus*	[24]
Acid tolerance	*Z. bailii*	[86, 91, 114, 115, 118, 126]
	*W. anomalus*	[115]
	*P. kudriavzevii*	[115, 125]

### Fermentation of sugars found in lignocellulosic feedstock: a key trait sought for in dung beetle yeasts

Yeasts are facultative anaerobes that can shift to fermentation in the absence of oxygen, a process that allows them to break down sugars, producing ethanol as a byproduct. Some yeasts termed as Crabtree positive yeasts can ferment even in the presence of oxygen [69]. They inhabit sugar-rich environments such as fruits in nature. Bioethanol production yeasts can either be acquired from culture collections or commercial suppliers, developed using classical genetic methods as well-developed evolutionary engineering or can be isolated from their natural environments. Yeasts from nature are most exploited in bioethanol production, due to their ability to utilize various fermentable sugars and convert them into ethanol. High ethanol productivity is an indispensable attribute for bioethanol production.

*Saccharomyces cerevisiae*, the ‘conventional’ yeast, an extensive model fermentation organism used for bioethanol production [3, 19, 70, 71] monopolised the bioethanol industry before the use of inexpensive and abundant lignocellulosic feedstock. Lignocellulosic feedstocks contain a wide range of sugars, which cannot be fermented by *S. cerevisiae*. One reason could be the ecology and niche preferences, since *S. cerevisiae* is a fruit sugar yeast whose niches in ripening and rotting fruits do not contain lignocellulosic sugars. It evolved to ferment a wide range of hexose sugars found in fruits and subsequently producing ethanol as a niche engineering strategy [2, 32, 72]. Its high ethanol tolerance made it the most employed yeast for industrial ethanol production. In addition to this attribute, its GRAS (generally regarded as a safe microorganism) status by the US FDA Organization, its genetic amenability, and its well-established systems-level attributes made it an ideal organism, which also accounts for its monopoly. Pentose sugars and sugar polymers represent a significant proportion of sugars in inexpensive and abundant lignocellulosic feedstocks [32]. Xylose, arabinose, glucose, galactose, and mannose are examples of key sugars present in lignocellulosic feedstocks [73–75]. The ability of yeasts to assimilate even a small amount of a variety of all available sugars enhances the productivity of the bioethanol production process and subsequently increases its economic feasibility [32]. Some researchers have reported several yeasts such as *Pichia* spp, *Candida* spp, *Brettanomyces spp*, *Scheffersomyces* spp, and others that can ferment xylose albeit at lower yields for sustainable production [46]. Cofermentation of key hexoses and pentoses has been touted as attractive in the reduction of the uneconomical fermentation time when pentoses are fermented, after the exhaustion of hexoses. This strategy is known to increase the economic feasibility of bioethanol [32]. Numerous approaches to enhance bioethanol production by the introduction of pentose pathways into yeasts strains via metabolic engineering have been reported [39, 40, 76–79]. Another approach would be to scout for yeasts that can utilize and ferment a wide range of sugars from nature. Such strains can be used as they are, or their genetic novelty can be used for reverse metabolic engineering of robust strains.

### Current sources of extremophilic yeasts for bioethanol fermentations

Yeasts with extremophilic traits are ideally important for the improvement of the efficiency of bioethanol production. Several extremophilic traits have been documented (Table 1). In general, stress tolerance is known among non-conventional yeasts. Some noticeable genera include *Saccharomyces spp* [15, 32, 80–82]*, Schizosaccharoymces spp* [15, 32]*, Dekkera spp* [15, 83–85]*, Pichia spp* [32], *Pachysolen spp* [15], and *Kluyveromyces spp* [80–82]. Thermotolerance is one of the most desirable characteristics of a bioethanol production strain. Alcoholic fermentation during simultaneous saccharification and fermentation is carried out at elevated temperatures. This decreases cooling costs, lowers the risk of contamination, and increases ethanol yields [32]. *Ogataea* (*Hansenula) polymorpha, Pichia kudriavzevii* (= *Issatchenkia orientalis* = *Candida krusei*), and *K. marxianus* are well-known thermotolerant yeasts [86–88]. *O polymorpha* is a methylotrophic yeast with thermotolerant traits, whose ability to ferment xylose is advantageous in bioethanol fermentation [89–91]. *P. kudriavzevii* although an opportunistic pathogen, has been identified as a multi-stress tolerant yeast that can be used for bioethanol production. It can tolerate elevated temperatures, high sugar concentrations, furan derivatives, and weak acids (e.g., acetic acid) (Table 1). Thermotolerant yeast, such as *K. marxianus* (Table 1)*,* can assimilate various sugars like xylose, cellobiose, lactose, and arabinose [91]. Osmotolerance is another desirable attribute of bioethanol fermenting yeasts. Non-conventional yeast species such as *Zygosaccharomyces rouxii* and *Zygosaccharomyces bailii* are known to possess outstanding osmotolerant abilities (Table 1), even though their ethanol production capacity is poor [32].

Other yeasts with beneficial traits have also been isolated. *Pichia stipitis, Candida shehatae,* and *Pachysolen tannophilus* have in common xylose fermentation abilities [16, 32, 92–94]. The recently described *Spathaspora passalidarum* also has xylose fermentation attributes [74, 90, 94–97].

### Sources of yeasts currently used in bioethanol production

Yeasts currently used for bioethanol fermentation have been isolated from different sources (Table 2). However, these yeast strains have not sufficiently addressed the current challenges of stream-lined carbon substrate utilization range, poor stress tolerance, and low ethanol productivity.

**Table 2:** Sources of potential yeasts for bioethanol production

Source	Yeast species	References
Wines	*D. bruxellensis*	[56, 99, 122, 127]
	*W. anomalus*	[128]
	*Pichia kluyveri*	[128, 129]
	*T. delbrueckii*	[128, 129]
Insects	*S. passalidarum*	[74, 95–97, 130, 131]
	*D. bruxellensis*	[54, 59]
	*K. marxianus*	[54]
	*O. polymorpha*	[54, 59, 132]
	*W. anomalus*	[132–134]
	*P. kudriavzevii*	[54, 133]
	*M. pulcherrima*	[58, 59, 133, 135]
Fruits	*M. pulcherrima*	[61, 135, 136]
	*P. kluyveri*	[136]
	*P. kudriavzevii*	[136]
Sugarcane juice	*P. kudriavzevii*	[137]
	*O. polymorpha*	[137]
	*K. marxianus*	[102]
Cocoa bean	*P. kudriavzevii*	[113, 138, 139]
	*P. kluyveri*	[139, 140]
Cheese	*P. kudriavzevii*	[113, 141]
	*K. marxianus*	[91, 141]
	*T. delbrueckii*	[141]
	*D. bruxellensis*	[142]
	*W. anomalus*	[141]

### Described methods and advances in isolation of yeasts from dung beetles

Dung beetles harbour yeasts in their guts in either a mutualistic or symbiotic relationship [143]. The precise roles of yeasts in specific sections of the dung beetle guts (foregut, midgut, and hindgut) could yield yeasts adapted to different niches whose potential in bioethanol production needs to be explored. Isolation of yeasts from dung beetles was extensively studied by [65]. Dung beetles are starved for some days to reduce the microbial populations as well as removing contaminating organisms [144, 145]. As a strategy to exclude non-resident surface microorganisms, beetles are initially surface disinfected with ethanol and rinsed with saline to wash off excess ethanol before dissection [65, 144–146]. After dissection, removal of guts is carried out aseptically before chopping them into pieces for isolation of resident yeasts. Alternatively, the legs, wings, and elytra can be removed before grinding the remaining body parts to isolate yeasts [66]. Use of homogenised gut contents as inoculum for selective isolation of yeasts with a specific phenotype using enrichment media supplemented with targeted compounds, such as preferred carbon sources, nitrogen sources, vitamin sources, and compounds supplying trace elements, or a specific stressor environment is common. As with the norm in isolation of yeasts, inhibition of growth of contaminating bacteria and moulds is carried out using antibiotics as well as using media with growth inhibitory compounds such as dichloran or biphenyl [147]. Culturing yeasts by simply streaking into an appropriate medium, without enrichment or after serial dilutions and subsequent plating of the suspensions into selective media agar [52, 144, 145], has been well documented to be successful. Single yeast colonies can then be isolated and verified using morphological, physiological, and biochemical tests. With the advent of DNA sequencing and sequence analysis technologies, the use of molecular approaches has become the most preferred for rapid and accurate identification of yeasts.

Yeasts can be selected for their abilities to consume different carbon sources by growing them on Yeast Extract Peptone Dextrose (YPD) media and substituting the dextrose with other carbon sources. Similarly, to select yeasts with abilities to tolerate different stresses, they can be grown in YPD media containing stressors (e.g., acetic acid, furfural, formic acid, and ethanol) of varying concentrations.

To check for the ability of yeasts to ferment a variety of sugars, they can be grown in fermentation media as described by [67], in test tubes containing Durham tubes and incubating at 30 °C for about 5 days. The presence of a gas bubble in the Durham tube will indicate that the yeast ferments the sugar [67].

### Conclusion and future of yeasts from dung beetles in producing bioethanol

Bioethanol has the potential to reduce greenhouse gas emissions. One noteworthy drawback of the petroleum fuel alternative is its economic feasibility due to the soaring costs of its production. One way to increase the feasibility of bioethanol development is a cost-effective bioprocess such as the utilization of inexpensive industrial and agro-industrial lignocellulosic feedstocks. However, there is a limited choice of robust yeasts with extreme traits needed to efficiently produce high ethanol titres for the feasibility of bioethanol commercialization. Due to the rich lignocellulose diet that dung beetles survive on, the isolation of specialised lignocellulosic degrading yeasts was proposed. Exploration of robust yeasts with novel traits from novel reservoirs needed to advance bioethanol production processes by reducing production costs, enhancing pre-treatment methods, and increasing ethanol yields is an attractive strategy that can be used to decrease greenhouse gas emissions. This augments the feasibility of conventional bioethanol production processes.

## Data Availability

Not applicable.
